# Supplementary material for: Castl: robust identification of spatially variable genes in spatial transcriptomics via an ensemble-based framework
Source: Brief Bioinform. 2026 Mar 6;27(2):bbag074. doi: 10.1093/bib/bbag074 (PMC12963980; doi:10.1093/bib/bbag074)
Supplement: bbag074_Castl_Supplementary_260131 [file bbag074_castl_supplementary_260131.docx]

**Castl: Robust Identification of Spatially Variable Genes in Spatial Transcriptomics via an** **Ensemble-based Framework**

Yiyi Yu^1,#^, Jiyuan Yang^2,#^, Ping-an He^1,*^ and Xiaoqi Zheng^3,4,5,*^

^1^ Department of Mathematics, College of Science, Zhejiang Sci-Tech University, Hangzhou, Zhejiang 310018, China

^2^ School of Mathematical Sciences, Shanghai Jiao Tong University, Shanghai 200240, China

^3^ Center for Single-Cell Omics, School of Public Health, Shanghai Jiao Tong University School of Medicine, Shanghai 200025, China

^4^ Hainan International Medical Center, Shanghai Jiao Tong University School of Medicine, Hainan 571400, China

^5^ The Guangxi Key Laboratory of Intelligent Precision Medicine, Guangxi Zhuang Autonomous Region, Nanning 530007, China

^#^These authors contributed equally

*Corresponding author: pinganhe@zstu.edu.cn (P.H.); xqzheng@shsmu.edu.cn (X.Z.).

[**Supplementary Tables**](#SupplementaryTables)

**[Table S1.](#TableS1)** [Analytical methods employed in this study.](#TableS1)

[**Table S2.** Simulation parameters for different spatial patterns.](#TableS2)

[**Table S3.** Simulation parameters for different statistical distribution.](#TableS3)

[**Table S4.** SRT datasets analyzed in this study.](#TableS4)

[**Table S5.** Quality scores of the top K SVGs identified by methods in the twelve DLPFC sample dataset.](#TableS5)

[**Table S6.** Quality scores of the top K SVGs identified by methods in mouse olfactory bulb datasets generated by different sequencing technologies.](#TableS6)

[**Supplementary Figures**](#SupplementaryFigures)

[**Figure S1.** Comparative evaluation of method performance across simulated spatial patterns.](#FigureS1)

[**Figure S2.** Comparative evaluation of method performance in simulated data with different statistical distributions.](#FigureS2)

[**Figure S3.** Comparative evaluation of method performance in simulated data with varying spot quantity scales.](#FigureS3)

[**Figure S4.** Comparative evaluation of method performance in simulated data with varying fold-change magnitudes.](#FigureS4)

[**Figure S5.** Comparative evaluation of method performance in simulated data with varying Gaussian noise intensities.](#FigureS5)

[**Figure S6.** Cell type clustering annotations for colorectal cancer and liver metastasis datasets.](#FigureS6)

[**Figure S7.** ROC curve analysis for colorectal cancer and liver metastasis datasets.](#FigureS7)

[**Figure S8.** Robustness analysis of the Castl ensemble framework.](#FigureS8)

**[Figure S9.](#FigureS9)** [Castl identifies cell-specific key SVGs in human colorectal cancer liver metastasis datasets.](#FigureS9)

**[Figure S10.](#FigureS10)** [Manual annotation of twelve DLPFC sections.](#FigureS10)

**[Figure S11.](#FigureS11)** [Castl enables cross-sample consistent identification of laminar SVGs in the human DLPFC dataset.](#FigureS11)

**[Figure S12.](#FigureS12)** [SPARK-X identifies many low-expression genes that lack spatial structure.](#FigureS12)

**[Figure S13.](#FigureS13)** [HeartSVG identifies many low-expression genes that lack spatial structure.](#FigureS13)

**[Figure S14.](#FigureS14)** [Tissue-specific enrichment analysis of methods in the human DLPFC dataset.](#FigureS14)

**[Figure S15.](#FigureS15)** [Detection consistency and functional specificity analysis of methods in the human DLPFC dataset.](#FigureS15)

**[Figure S16.](#FigureS16)** [Spatial autocorrelation analysis of top-K SVGs identified by methods in the human DLPFC dataset.](#FigureS16)

[**Figure S17.** Clustering performance using SVGs identified by different methods.](#FigureS17)

**[Figure S18.](#FigureS18)** [FDPp-Agg enhances the identification of method-specific SVGs.](#FigureS18)

**[Figure S19.](#FigureS19)** [Castl enables cross-technology consistent identification of laminar SVGs in the mouse olfactory bulb dataset.](#FigureS19)

**[Figure S20.](#FigureS20)** [KEGG enrichment comparison analysis of methods in the mouse olfactory bulb dataset.](#FigureS20)

**[Figure S21.](#FigureS20)** [Detection consistency and functional specificity analysis of methods in the mouse olfactory bulb dataset.](#FigureS20)

**[Figure S22.](#FigureS22)** [Tissue-specific enrichment analysis of methods in the mouse olfactory bulb dataset.](#FigureS22)

**[Figure S23.](#FigureS23)** [Upset plots of SVGs identified by different detection methods in the Mouse Hypothalamic Preoptic Region dataset generated by MERFISH.](#FigureS23)

**[Figure S24.](#FigureS24)** [FDPp-Agg enables cell-type-specific SVGs in the mouse Hypothalamic Preoptic Region dataset.](#FigureS24)

**Supplementary Notes**

We generated comprehensive simulated datasets incorporating diverse typical spatial patterns characterized by different positional distributions and proportion of expressing spots, various distributions (Posi, ZIP, NB, ZINB), different proportions of SVGs among all detected genes (10%, 30%, 50%, 70%, 90%), distinct spot quantity scales (1000, 1500, 3000, 5000, 8000, 10000 spots), multiple fold changes (2$\times$, 4$\times$, 6$\times$, 8$\times$, 10$\times$), varying Gaussian noise intensities (0.00, 0.01, 0.05, 0.1, 0.2, 0.3, 0.4). These datasets were designed to simulate complex scenarios for systematic performance validation of the methods, with parameter settings referencing those of SPARK and HeartSVG.

Specifically, to introduce Gaussian noise into the generated simulated data, we first normalized the original count matrix $X$ by linearly scaling it to the interval $[0,1]$, thereby eliminating the impact of expression magnitude disparities on noise addition. Gaussian noise $\epsilon\sim\mathcal{N}(0,\sigma^{2})$ is then added to the normalized data, where $\sigma$, representing noise level parameter, follows the parameter settings of HeartSVG to control technical variation intensity. The data were subsequently threshold-clipped to maintain biological plausibility and inverse-normalized to restore the original expression scale.

**Supplementary Tables**

**Table S1. Analytical methods employed in this study.**

| **Method** | **Core Methodology** | **Language** | **Statistical Index** | **Refs.** |
| --- | --- | --- | --- | --- |
| SpatialDE | Gaussian Process | Python | *q*-value | [1] |
| SPARK | Generalized Linear Spatial Model | R | adjusted *p*-value | [2] |
| SPARK-X | Covariance Test | R | adjusted *p*-value | [3] |
| SOMDE | Self-Organizing Map and Gaussian Process | Python | *q*-value | [4] |
| SpaGCN | Graph Convolutional Network | Python | adjusted *p*-value | [5] |
| Spanve | Kullback-Leibler Divergence | Python | FDR | [6] |
| HeartSVG | Kernel Half-Convolution Process | R | adjusted *p*-value | [7] |

**Table S2. Simulation parameters for different spatial patterns.**

| **Category** | **Pattern** | **PropSpots** |
| --- | --- | --- |
| Focal | Hotspot | 6.3% |
| Continuous | Gradient | 74.8% |
| Linear | Streak | 5.3% |
|  | Streaks | 14.1% |
|  | Curve | 13.6% |
| Concentric | Ring | 17.6% |
|  | Rings | 23.6% |
| Structured | Pattern I | 18.2% |
|  | Pattern II | 38.0% |
|  | Pattern III | 20.7% |

**Table S3. Simulation parameters for different statistical distribution.**

|  | **Higher Signal (SVG)** | | | **Lower Signal (SVG)** | | | **Noise (nSVG)** | | |
| --- | --- | --- | --- | --- | --- | --- | --- | --- | --- |
|  | Mean ($\mu$) | Dispersion ($\theta$) | Zero% ($\pi_{0}$) | Mean ($\mu$) | Dispersion ($\theta$) | Zero% ($\pi_{0}$) | Mean ($\mu$) | Dispersion ($\theta$) | Zero% ($\pi_{0}$) |
| **Posi** | 2$\times$10 | - | - | 2/10 | - | - | 2 | - | - |
| **ZIP** | 2$\times$10 | - | 0.05 | 2/10 | - | 0.05 | 2 | - | 0.05 |
| **NB** | 2$\times$10 | 5 | - | 2/10 | 5 | - | 2 | 5 | - |
| **ZINB** | 2$\times$10 | 5 | 0.05 | 2/10 | 5 | 0.05 | 2 | 5 | 0.05 |

**Table S4. SRT datasets analyzed in this study.**

| **Dataset** | **Platform** | **Section** | **Dimensions** | **Refs.** |
| --- | --- | --- | --- | --- |
| Human Colorectal Cancer | 10x Visium | Colon1 | 3,313 spots and 36,601 genes | [8] |
|  |  | Colon2 | 4,174 spots and 36,601 genes |  |
|  |  | Colon3 | 4,007 spots and 36,601 genes |  |
|  |  | Colon4 | 3,902 spots and 36,601 genes |  |
| Human Liver Cancer | 10x Visium | Liver1 | 3,826 spots and 36,601 genes | [8] |
|  |  | Liver2 | 4,658 spots and 36,601 genes |  |
|  |  | Liver3 | 3,695 spots and 36,601 genes |  |
|  |  | Liver4 | 3,721 spots and 36,601 genes |  |
| Human Dorsolateral Prefrontal Cortex | 10x Visium | 151507 | 4,226spots and 33,538genes | [9, 10] |
|  |  | 151508 | 4,384spots and 33,538genes |  |
|  |  | 151509 | 4,789spots and 33,538genes |  |
|  |  | 151510 | 4,634spots and 33,538genes |  |
|  |  | 151669 | 3,661spots and 33,538genes |  |
|  |  | 151670 | 3,498spots and 33,538genes |  |
|  |  | 151671 | 4,110spots and 33,538genes |  |
|  |  | 151672 | 4,015spots and 33,538genes |  |
|  |  | 151673 | 3,639spots and 33,538genes |  |
|  |  | 151674 | 3,673spots and 33,538genes |  |
|  |  | 151675 | 3,592spots and 33,538genes |  |
|  |  | 151676 | 3,460spots and 33,538genes |  |
| Mouse Olfactory Bulb | Stereo-seq | - | 19,109spots and 27,106genes | [11,  12] |
| Mouse Olfactory Bulb | Slide-seqV2 | Puck_200127_15 | 21,724spots and 21,217genes | [13] |
| Mouse Hypothalamic Preoptic Region | MERFISH | - | 4975spots and 160genes | [14, 15] |

**Table S5. Quality scores of the top K SVGs identified by methods in the twelve DLPFC sample dataset.**

| **Method** | **Top K** | | | | | |
| --- | --- | --- | --- | --- | --- | --- |
|  | **1000** | **2000** | **3000** | **4000** | **5000** | **6000** |
| SpatialDE | 0.6719 | 0.5913 | 0.5799 | 0.5815 | 0.5578 | 0.5280 |
| SPARK | 0.5573 | 0.5280 | 0.5241 | 0.5230 | 0.5340 | 0.5116 |
| SPARK-X | **0.8041** | **0.7962** | **0.7723** | 0.7536 | 0.7417 | 0.7080 |
| SOMDE | 0.4653 | 0.5044 | 0.4858 | 0.4515 | 0.4007 | 0.3720 |
| SpaGCN | 0.1279 | 0.1185 | 0.1185 | 0.1185 | 0.1185 | 0.1185 |
| Spanve | 0.7686 | 0.7099 | 0.6517 | 0.5902 | 0.5268 | 0.4670 |
| HeartSVG | 0.4593 | 0.3230 | 0.361 | 0.2866 | 0.2324 | 0.2170 |
| Rank-Agg | 0.6789 | 0.6414 | 0.6230 | 0.6050 | 0.5924 | 0.5710 |
| Pval-Agg | 0.7318 | 0.5989 | 0.5906 | 0.5609 | 0.5364 | 0.5322 |
| FDPp-Agg | 0.7642 | 0.7806 | 0.7585 | **0.7606** | **0.7619** | **0.7609** |

**Table S6. Quality scores of the top K SVGs identified by methods in mouse olfactory bulb datasets generated by different sequencing technologies.**

| **Method** | **Top K** | | | | | |
| --- | --- | --- | --- | --- | --- | --- |
|  | **150** | **300** | **450** | **600** | **750** | **900** |
| SpatialDE | NA | NA | NA | NA | NA | NA |
| SPARK | 0.6861 | 0.6255 | 0.6206 | 0.6085 | 0.5824 | 0.5616 |
| SPARK-X | 0.6658 | 0.6288 | 0.6108 | 0.5965 | 0.5763 | 0.5564 |
| SOMDE | 0.6026 | 0.6259 | 0.5593 | 0.5506 | 0.5391 | 0.5423 |
| SpaGCN | 0.5104 | 0.5104 | 0.5104 | 0.5104 | 0.5104 | 0.5104 |
| Spanve | 0.5046 | 0.5046 | 0.5046 | 0.5046 | 0.5046 | 0.5046 |
| HeartSVG | 0.7936 | **0.8217** | 0.6284 | 0.4978 | 0.4099 | 0.3227 |
| Rank-Agg | **0.8477** | 0.7442 | 0.7058 | 0.6446 | 0.6141 | 0.5861 |
| Pval-Agg | 0.7754 | 0.6950 | 0.6201 | 0.5634 | 0.5499 | 0.5270 |
| FDPp-Agg | 0.8463 | 0.7417 | **0.7279** | **0.7175** | **0.7302** | **0.6684** |

**Supplementary Figures**


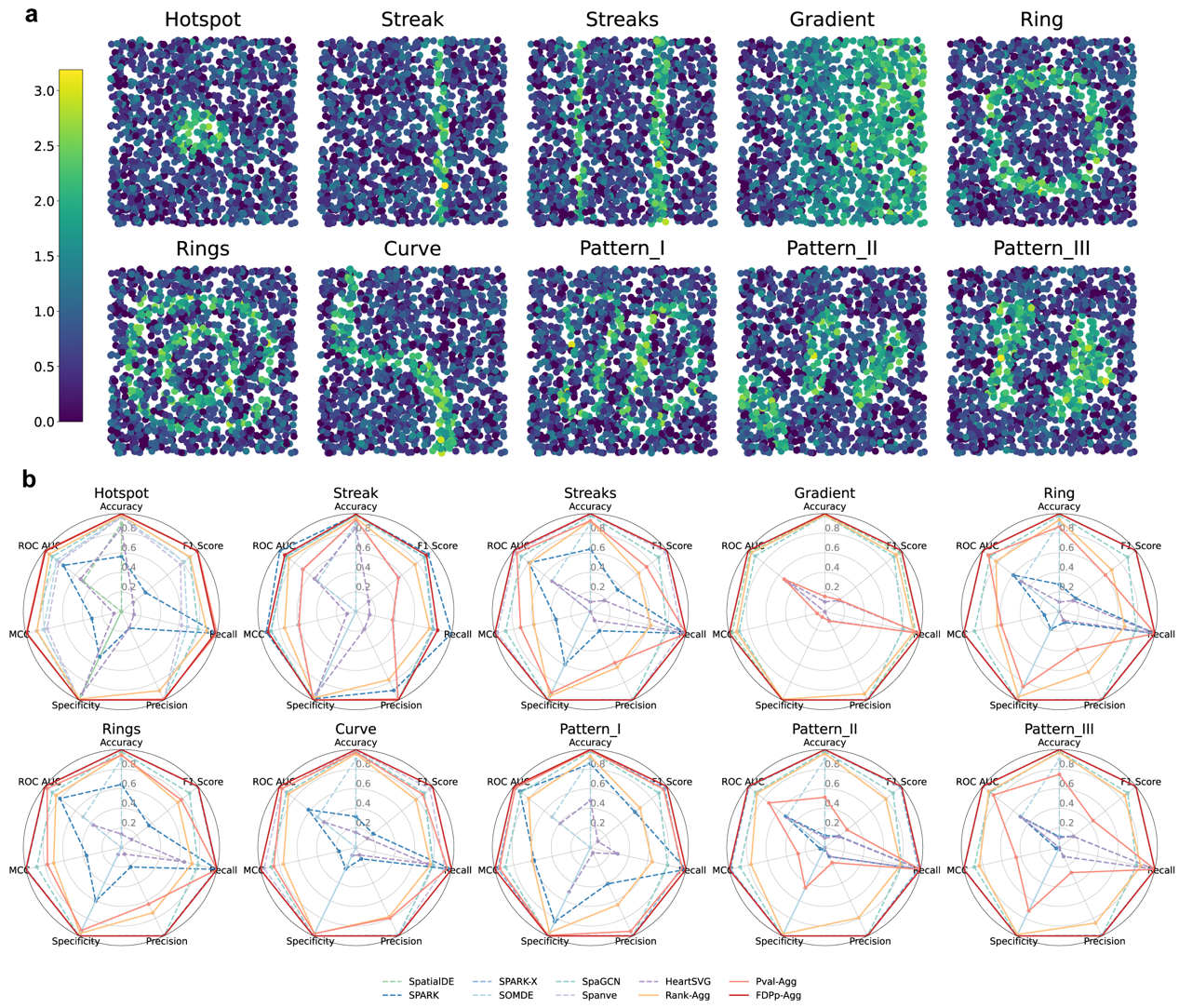


**Figure S1. Comparative evaluation of method performance across simulated spatial patterns. a** Visualization of simulated gene expression across ten spatial patterns. **b** Radar chart illustrating seven comprehensive metrics for seven benchmark methods and three consensus methods.


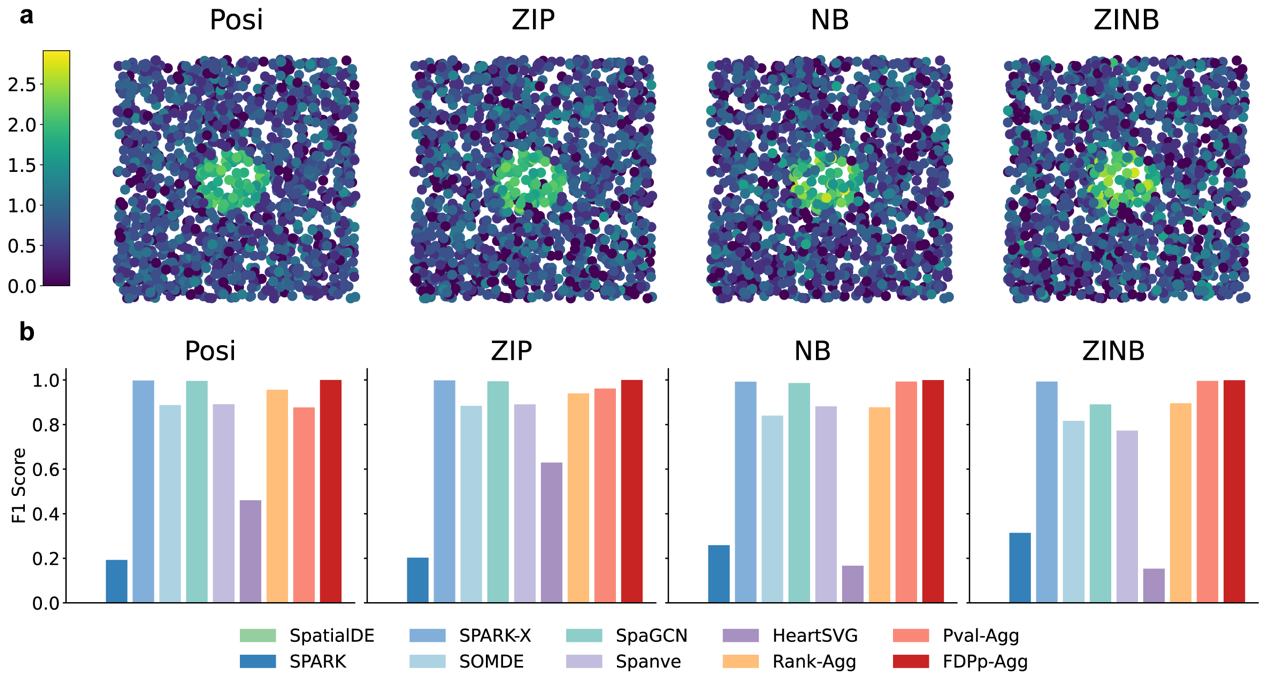


**Figure S2. Comparative evaluation of method performance in simulated data with different statistical distributions. a** Visualization of simulated gene expression across four statistical distributions. **b** Histogram plots depict F1 score comparisons (*y*-axis) for all evaluated methods across different statistical distributions.


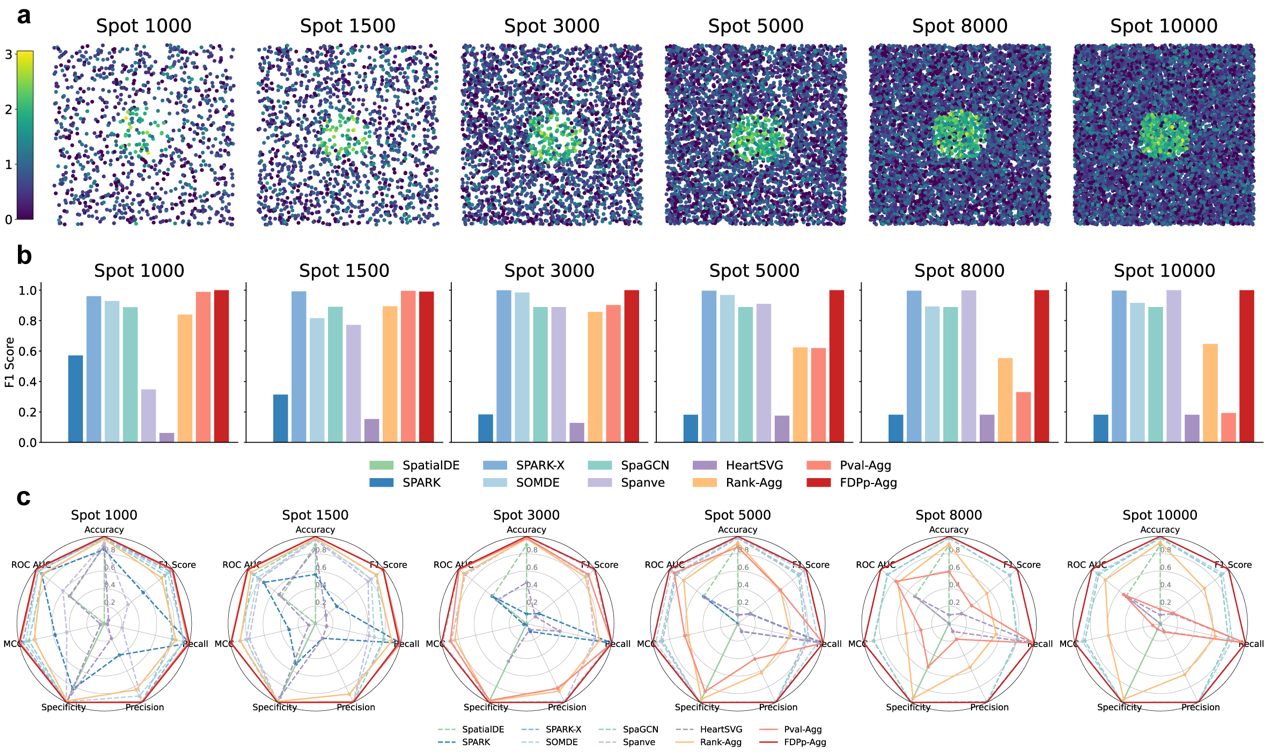


**Figure S3. Comparative evaluation of method performance in simulated data with varying spot quantity scales. a** Visualization of simulated gene expression patterns across different numbers of spots. **b** Histogram plots depict F1 score comparisons (*y*-axis) for all evaluated methods across different numbers of spot quantities. **c** Radar plots illustrate the comparison of seven comprehensive metrics for all evaluated methods across different numbers of spots.


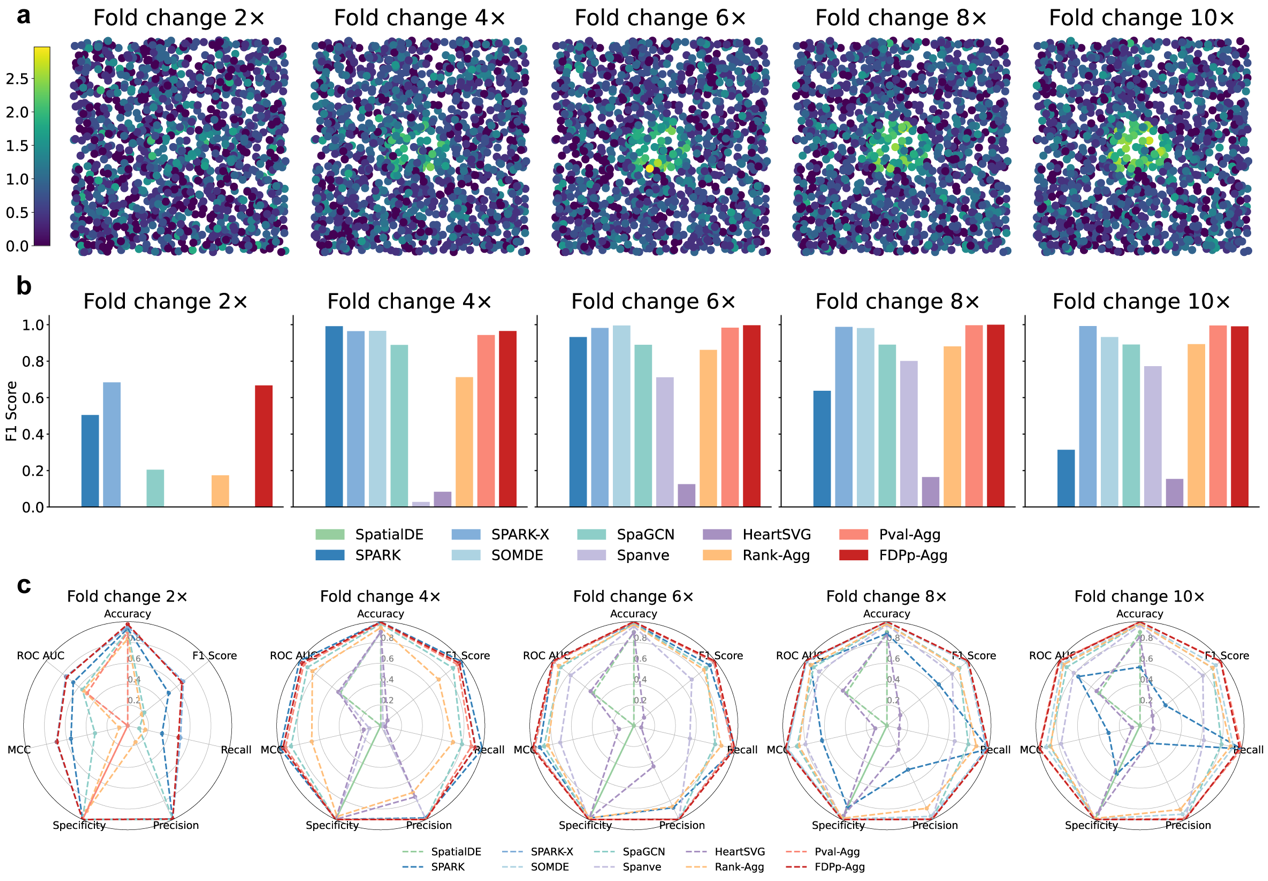


**Figure S4. Comparative evaluation of method performance in simulated data with varying fold-change magnitudes. a** Visualization of simulated gene expression across different fold-change magnitudes. **b** Histogram plots depict F1 score comparisons (*y*-axis) for all evaluated methods across varying fold-change magnitudes. **c** Radar plots illustrate the comparison of seven comprehensive metrics for all evaluated methods across different fold-change magnitudes.


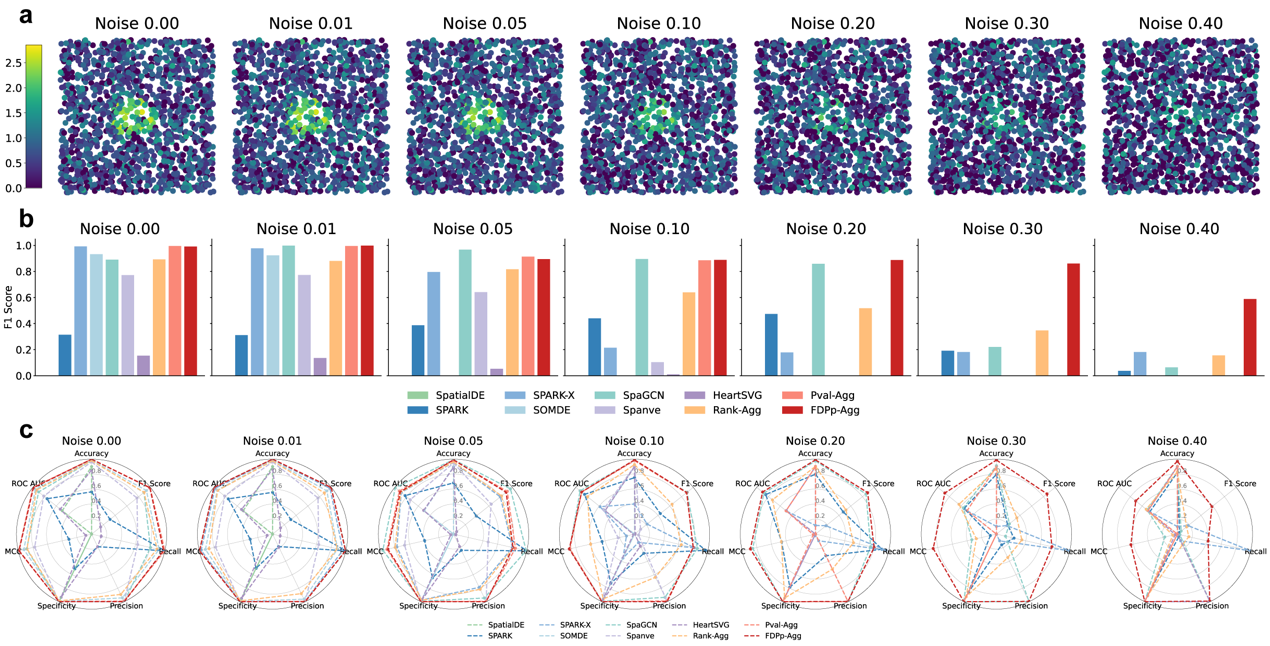


**Figure S5. Comparative evaluation of method performance in simulated data with varying Gaussian noise intensities.** **a** Visualization of simulated gene expression across different Gaussian noise intensities. **b** Histogram plots depict F1 score comparisons (*y*-axis) for all evaluated methods across varying Gaussian noise intensities. **c** Radar plots illustrate the comparison of seven comprehensive metrics for all evaluated methods across different Gaussian noise intensities.


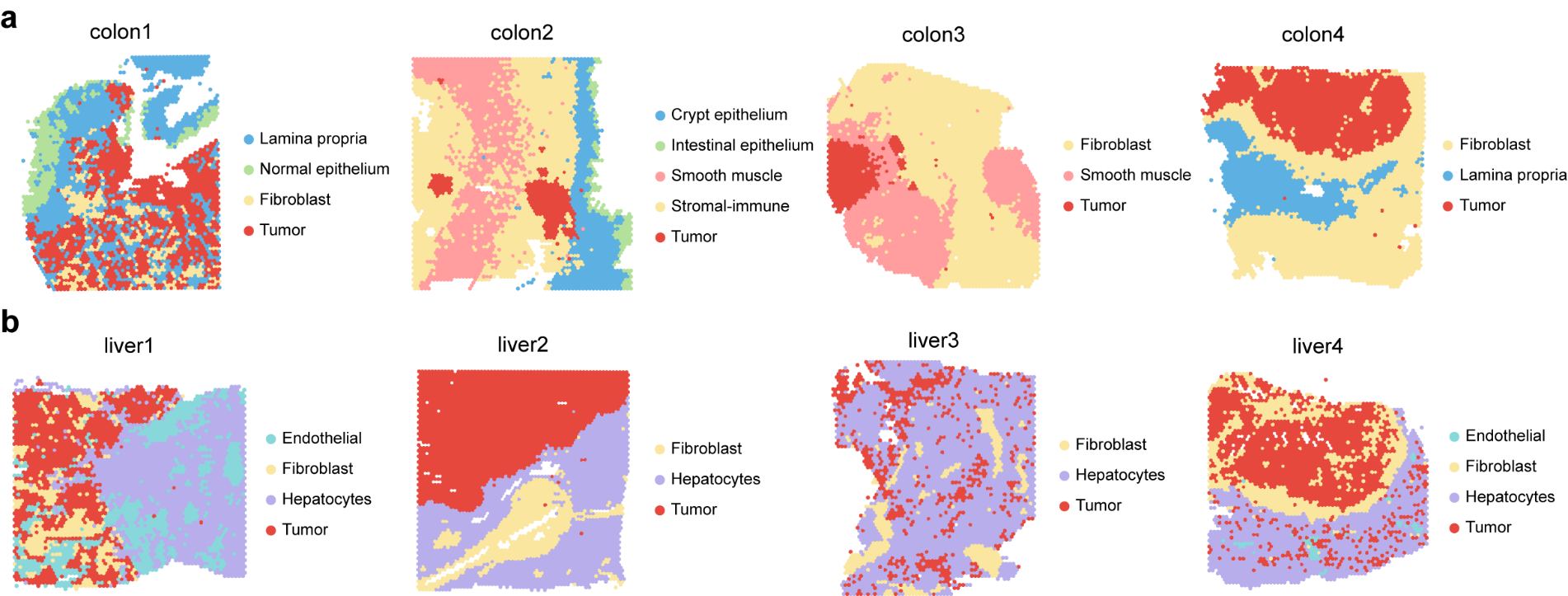


**Figure S6. Cell type clustering annotations for colorectal cancer and liver metastasis datasets.** Unsupervised spatial clustering results of colorectal cancer (**a**) and liver metastasis (**b**) datasets, with cluster annotations referenced to the studies by Wu et al.


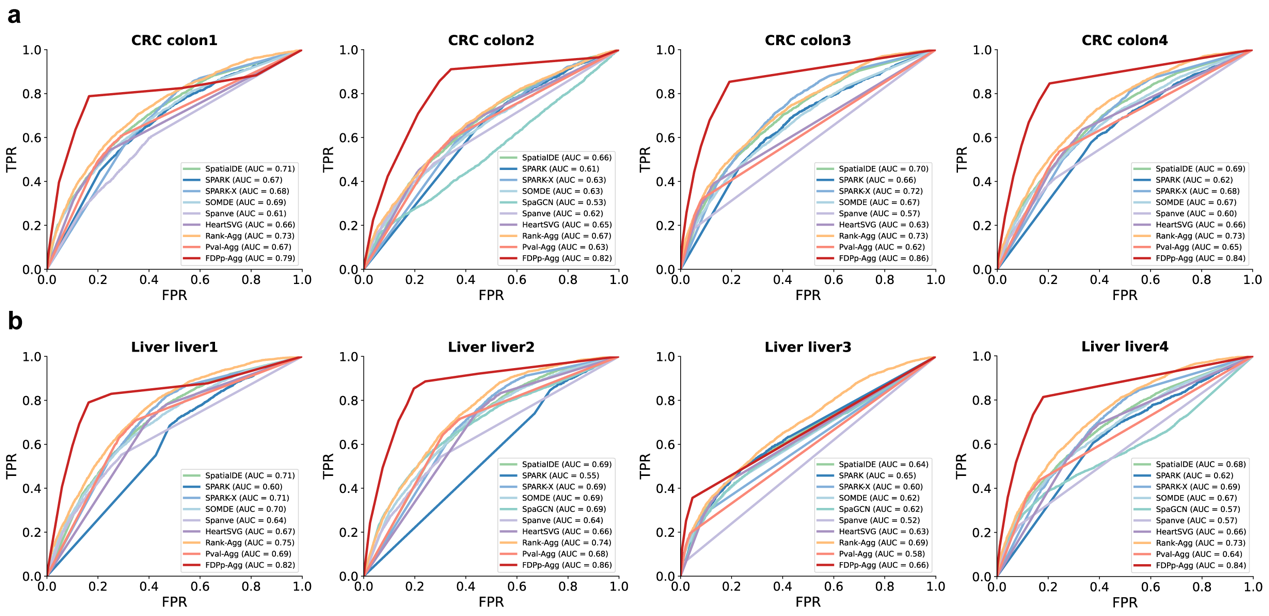


**Figure S7. ROC curve analysis for colorectal cancer and liver metastasis datasets.** ROC curves depicting true positive rate (TPR) vs false positive rate (FPR) for colorectal cancer (**a**) and liver metastasis (**b**) datasets, evaluated using subtype gene sets from the Colorectal Cancer Subtyping Consortium (CRCSC) as reference.


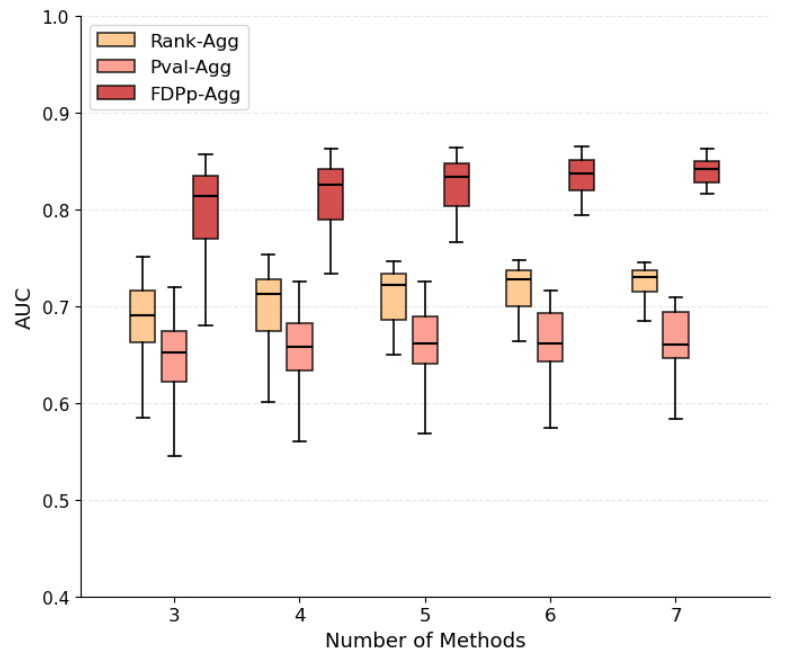


**Figure S8. Robustness analysis of the Castl ensemble framework. a** Box plots showing the distribution of AUC values (y-axis) for the three parallel ensemble methods across different subsets of baseline methods (x-axis). Performance was assessed on eight colorectal cancer liver metastasis samples using consensus molecular biomarkers from the CRCSC as ground truth.


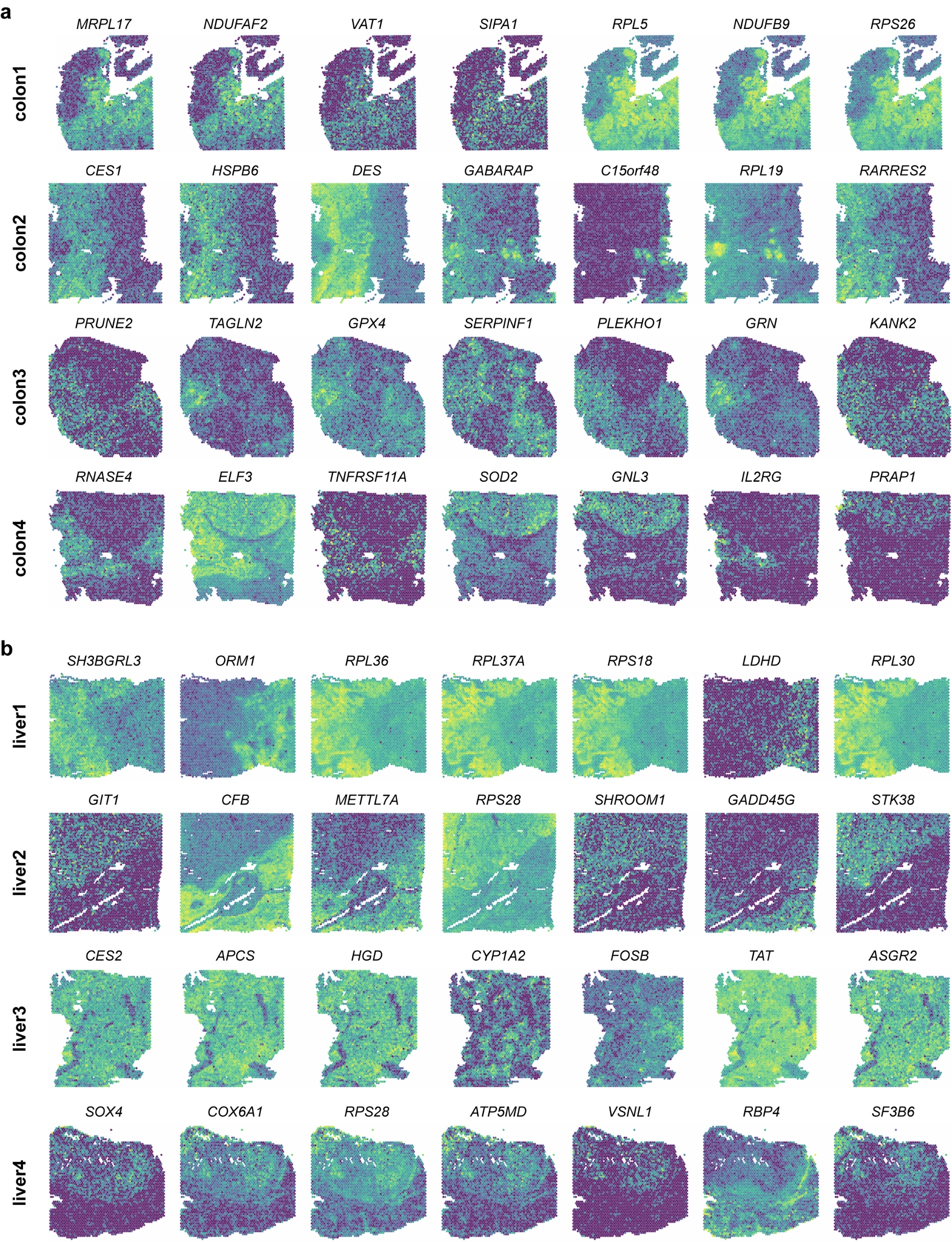


**Figure S9. Castl identifies cell-specific key SVGs in human colorectal cancer liver metastasis datasets.** Visualization of gene expression for cell-specific SVGs identified by Castl, spanning four colon sections (**a**) and four liver sections (**b**). Each panel represents a single gene.


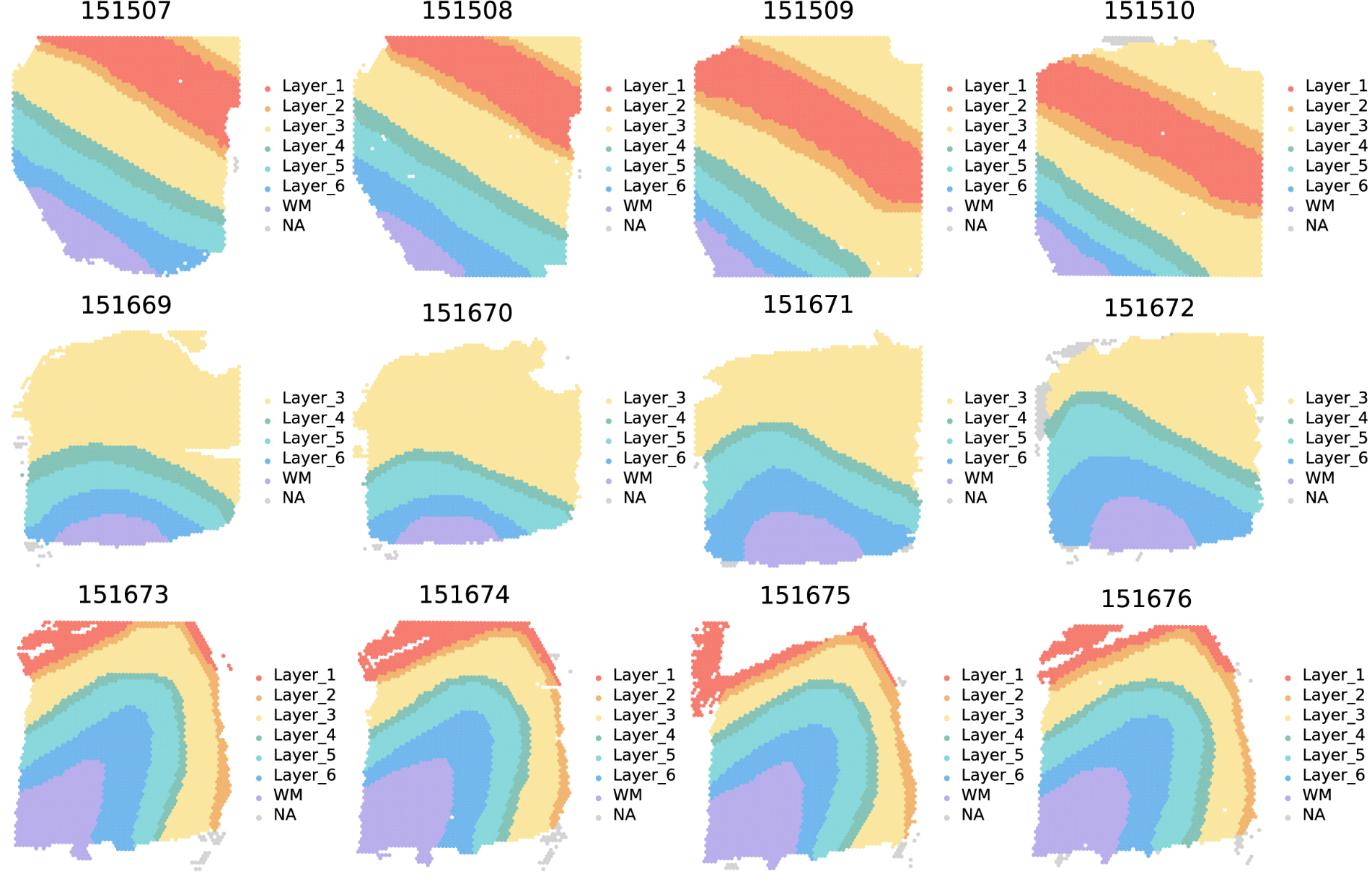


**Figure S10. Manual annotation of twelve DLPFC sections.**


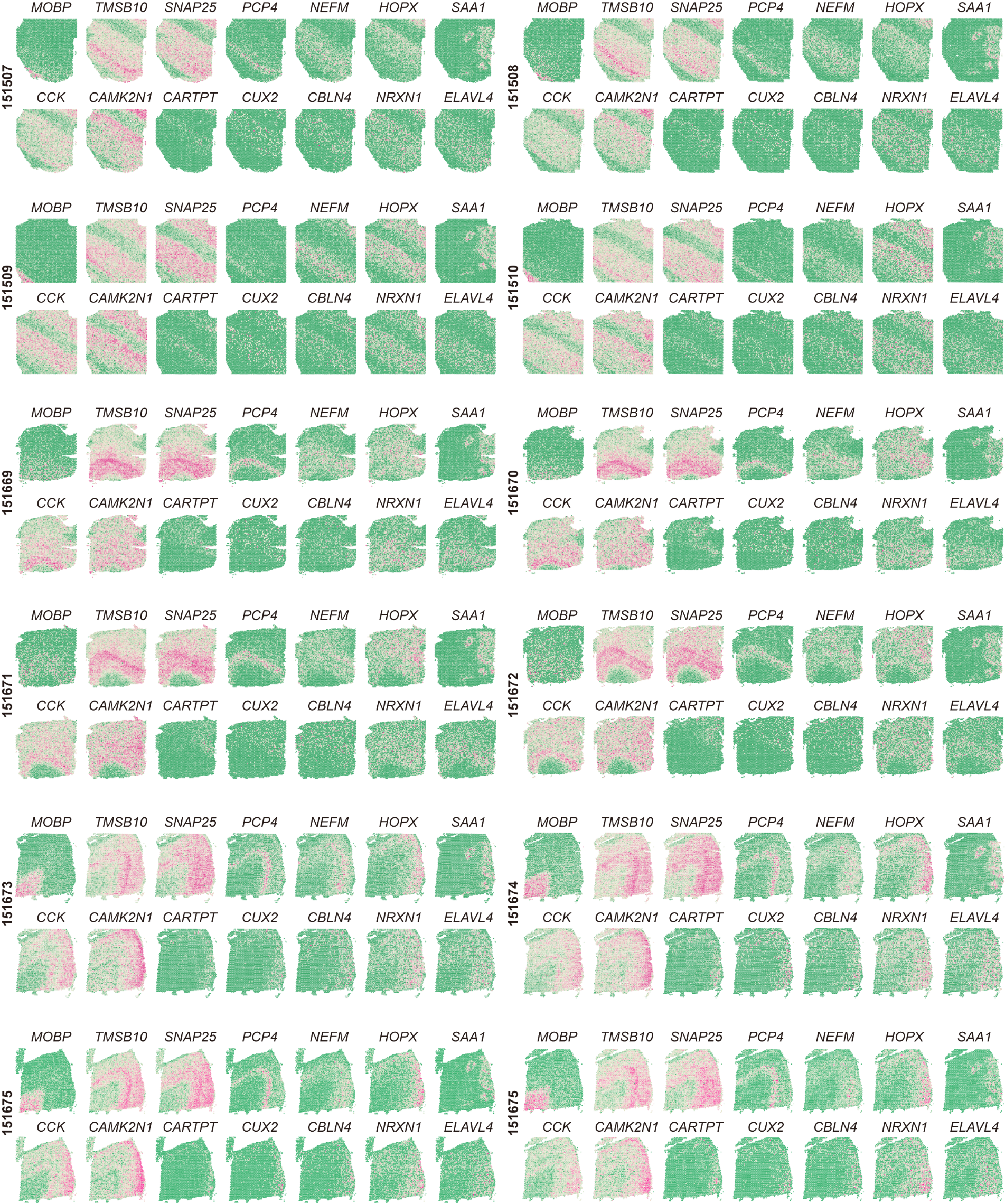


**Figure S11. Castl enables cross-sample consistent identification of laminar SVGs in the human DLPFC dataset.** Visualization of gene expression for SVGs with significant laminar organization consistently identified by Castl across twelve DLPFC sections. Each panel represents a gene.


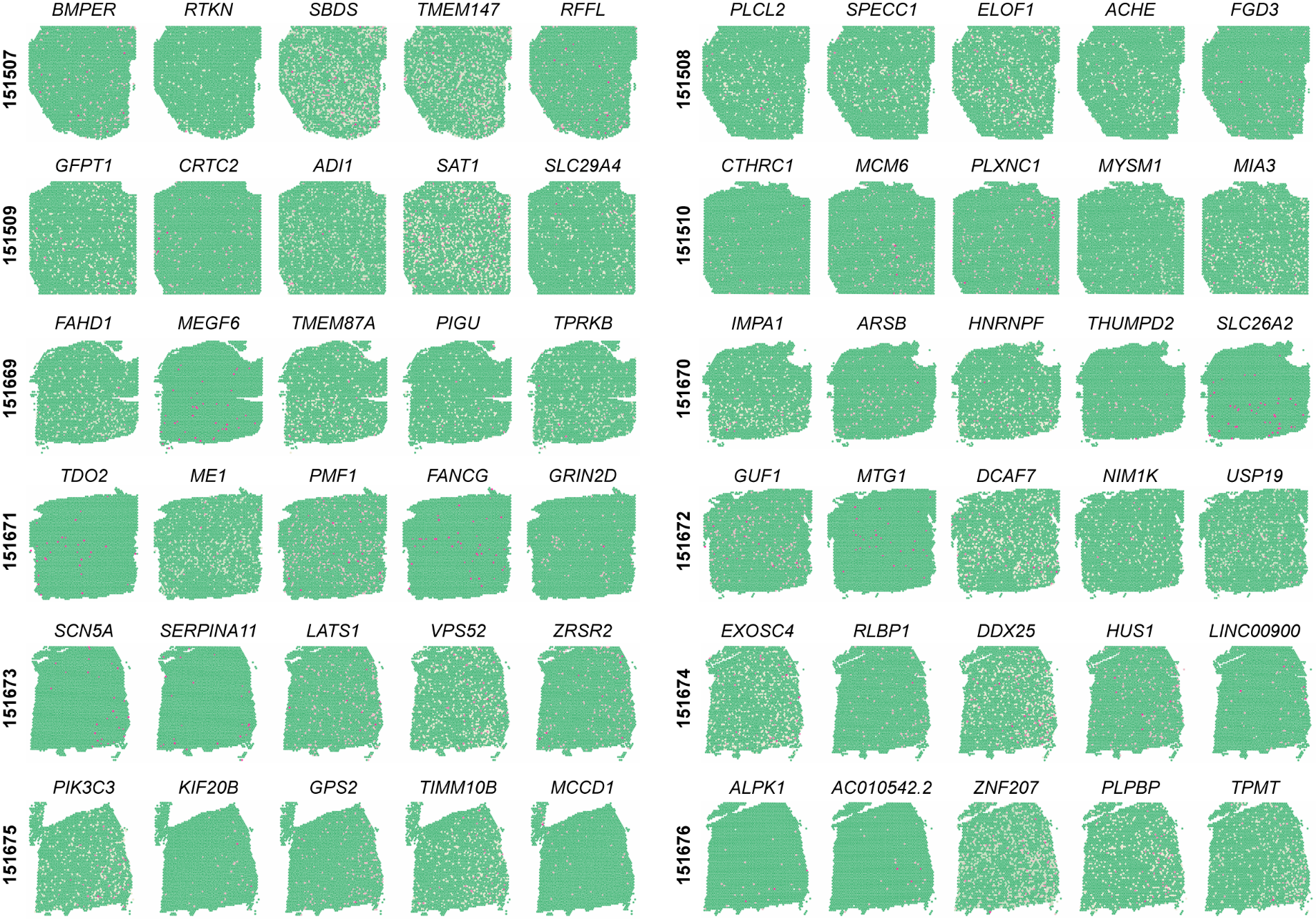


**Figure S12. SPARK-X identifies many low-expression genes that lack spatial structure.** Visualization of gene expression for SVGs identified by SPARK-X across twelve DLPFC sections. Each panel represents a gene.


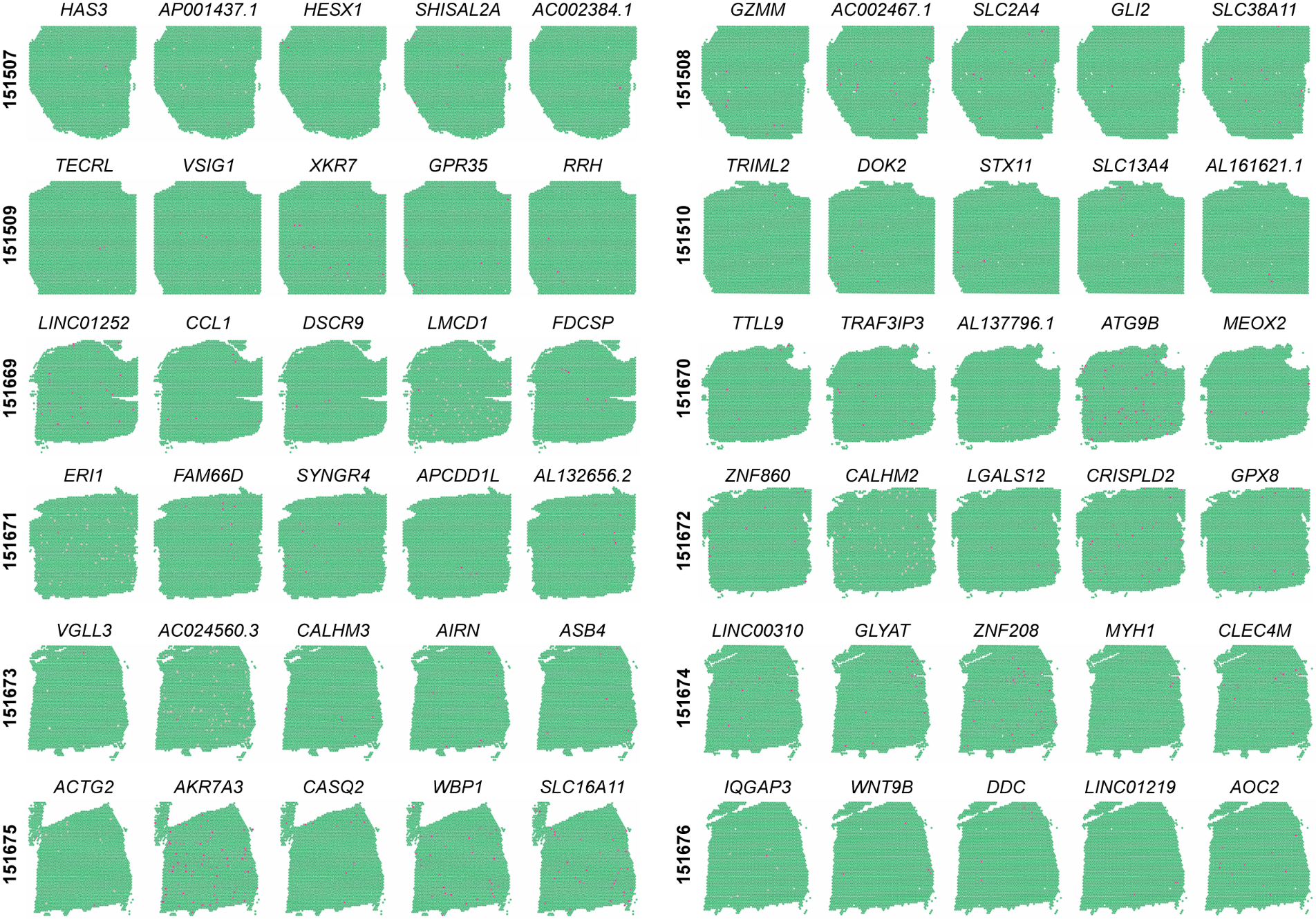


**Figure S13. HeartSVG identifies many low-expression genes that lack spatial structure.** Visualization of gene expression for SVGs identified by HeartSVG across twelve DLPFC sections. Each panel represents a gene.


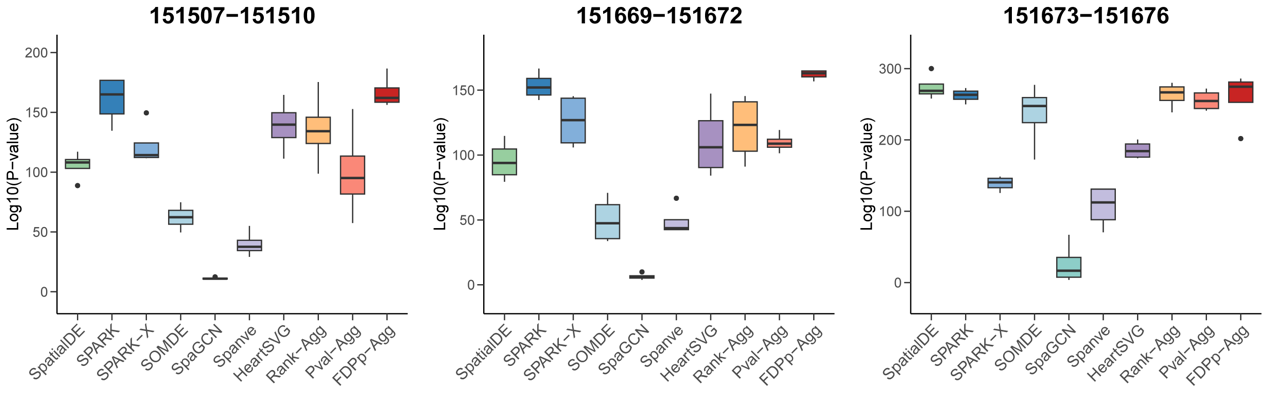


**Figure S14. Tissue-specific enrichment analysis of methods in the human DLPFC dataset.** The box plots depicting the enrichment significance (*y*-axis: -log10(*p*-value)) of SVGs identified by different methods in Cerebral Cortex tissue across the same group of DLPFC sections. The center line, box limits, and whiskers respectively depict the median, upper and lower quartiles, and 1.5$\times$ interquartile range.


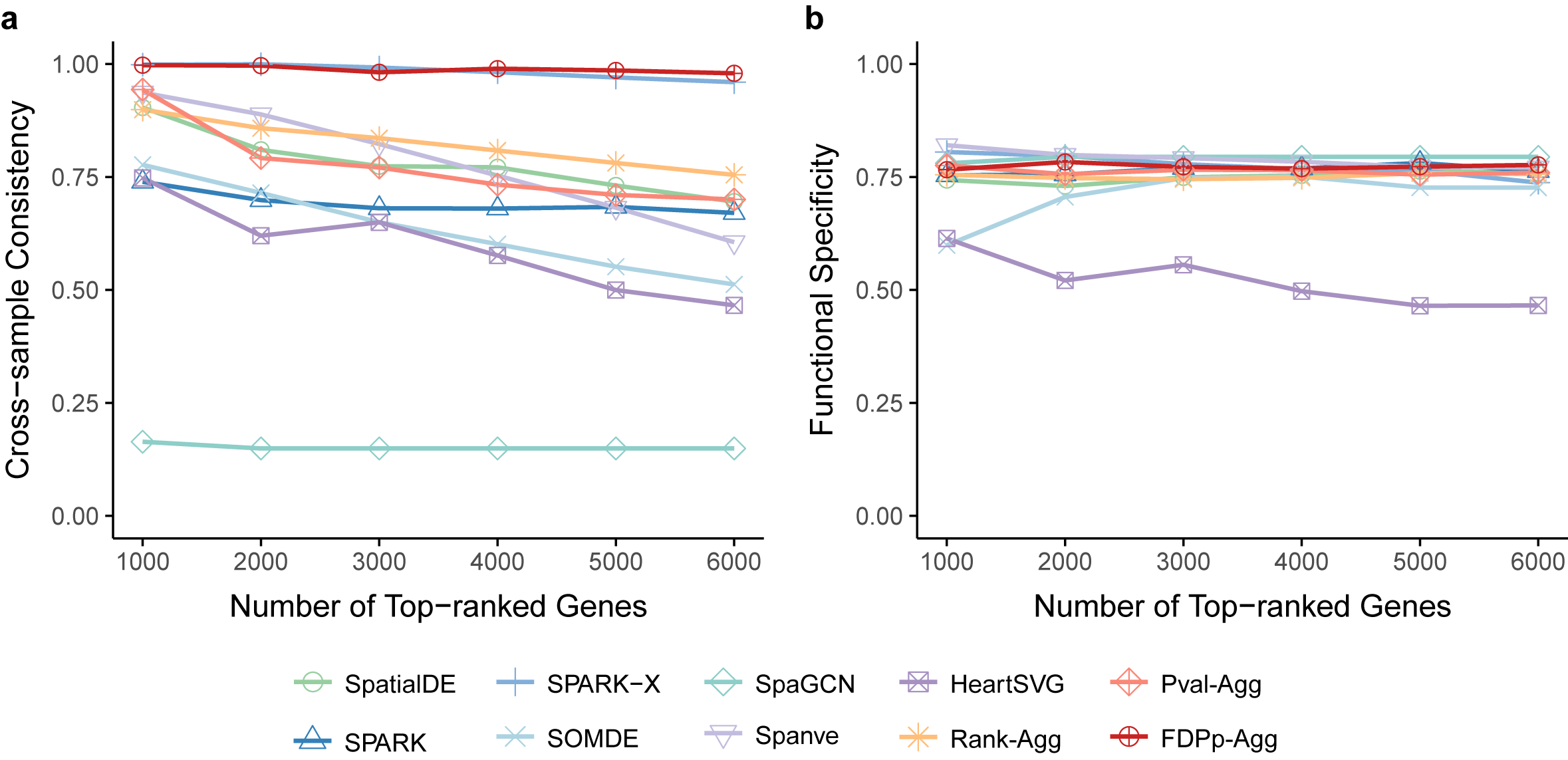


**Figure S15. Detection consistency and functional specificity analysis of methods in the human DLPFC dataset. a** The line plots illustrate the consistency (*y*-axis; see Methods for details) of detection methods across the twelve DLPFC samples for the top 1000 to 6000 SVGs (*x*-axis), using all identified SVGs when the number exceeds the method’s detection quantity. **b** The line plots depict the proportion of enriched genes (*y*-axis) among the top 1000 to 6000 SVGs (*x*-axis) identified by each detection method across the twelve DLPFC samples.


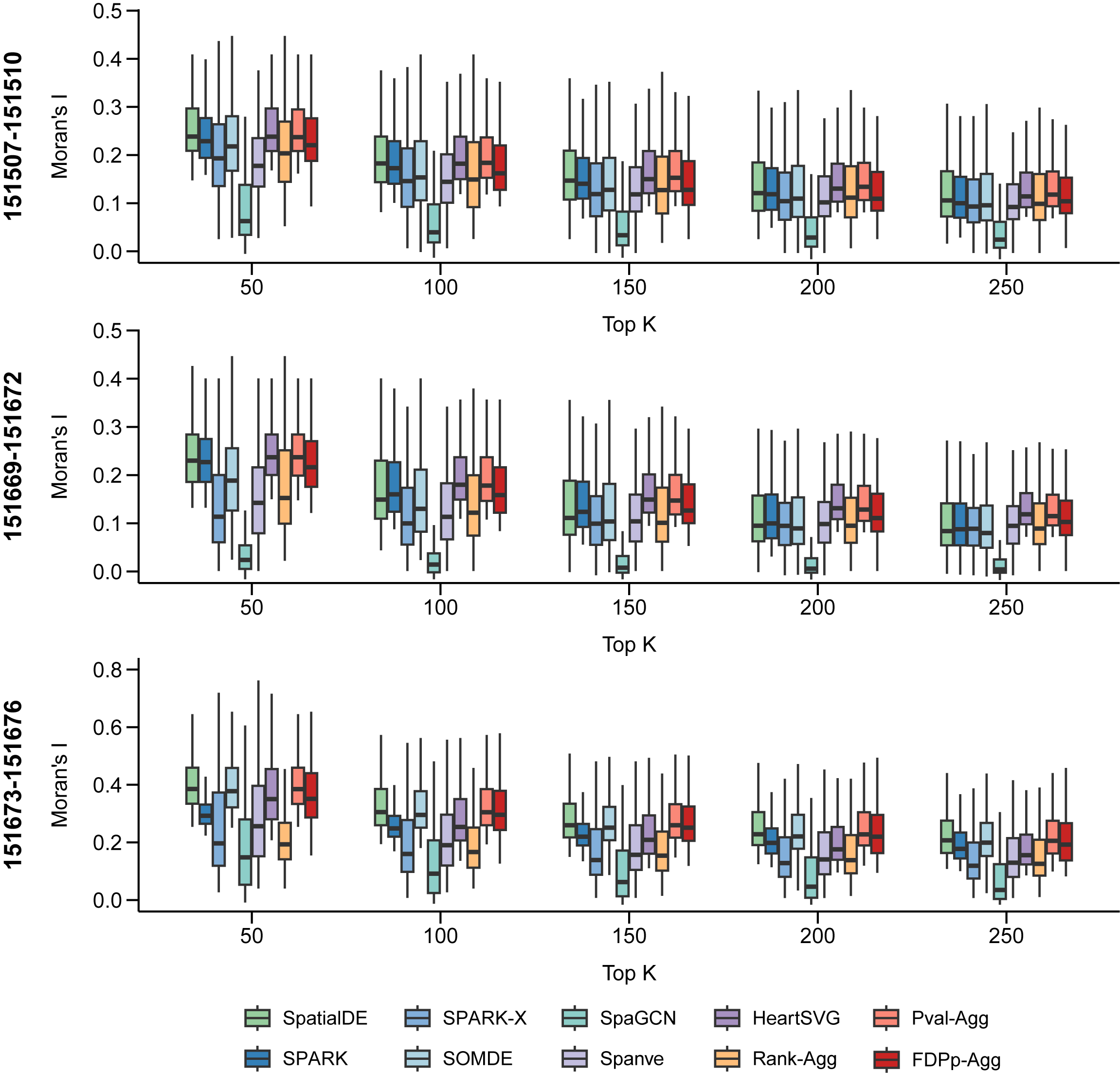


**Figure S16. Spatial autocorrelation analysis of top-K SVGs identified by methods in the human DLPFC dataset.** The box plots display Moran’s I index values (*y*-axis) of top-K genes (*x*-axis) identified by different methods across the same group of DLPFC sections. The center line, box limits, and whiskers respectively depict the median, upper and lower quartiles, and 1.5$\times$ interquartile range.


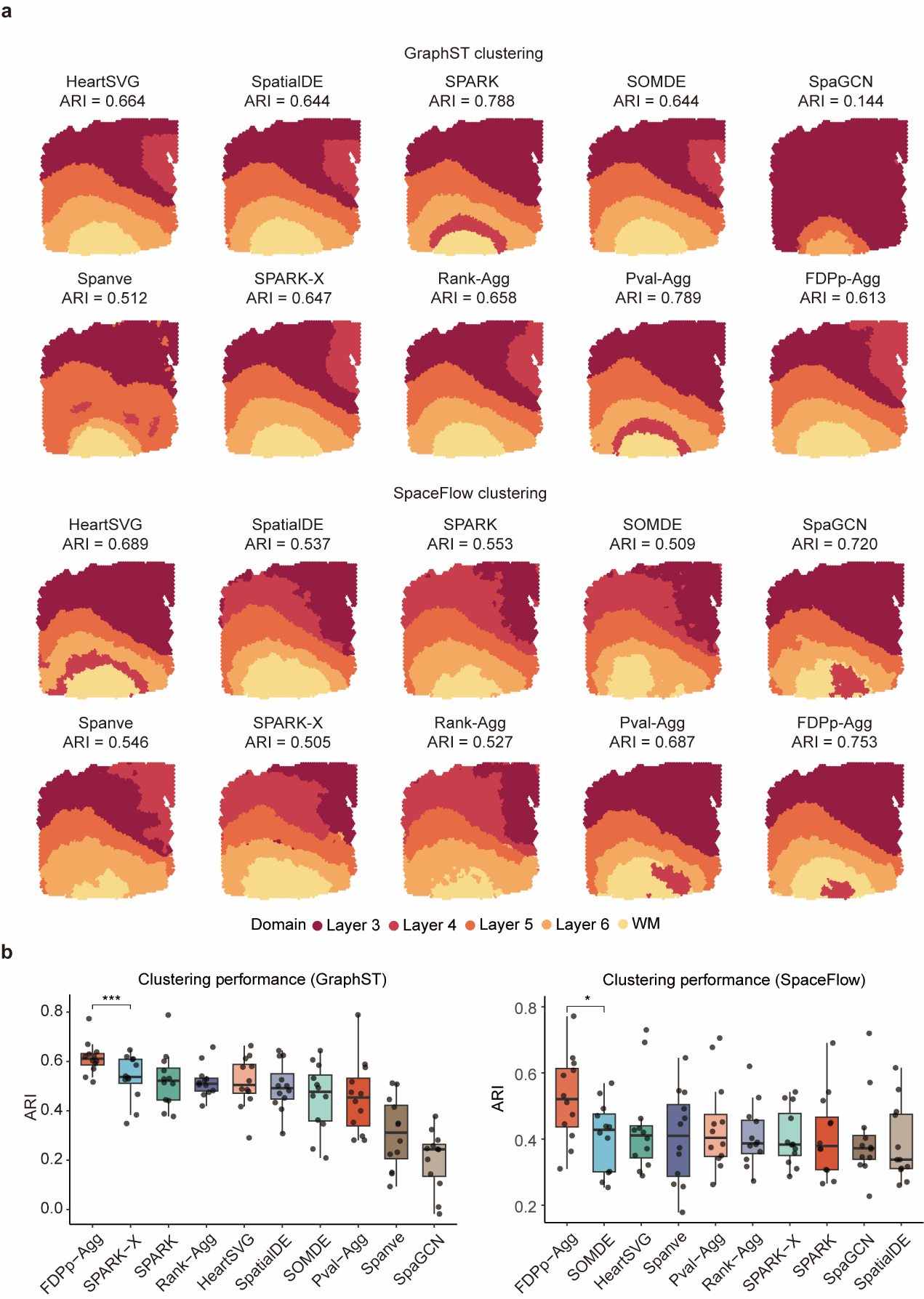


**Figure S17. Clustering performance using SVGs identified by different methods. a** Spatial domain detection results by different methods on the sample 151672 of the DLPFC dataset. **b** Boxplots show the Adjusted Rand Index (*y*-axis) achieved by GraphST (left) and SpaceFlow (right) based on SVGs identified by each baseline or ensemble method (*x*-axis), evaluated against manual tissue annotations across twelve DLPFC sections. Statistical significance was determined using a one-sided paired Wilcoxon signed-rank test (* P < 0.05, ** P < 0.01, *** P < 0.001).

**
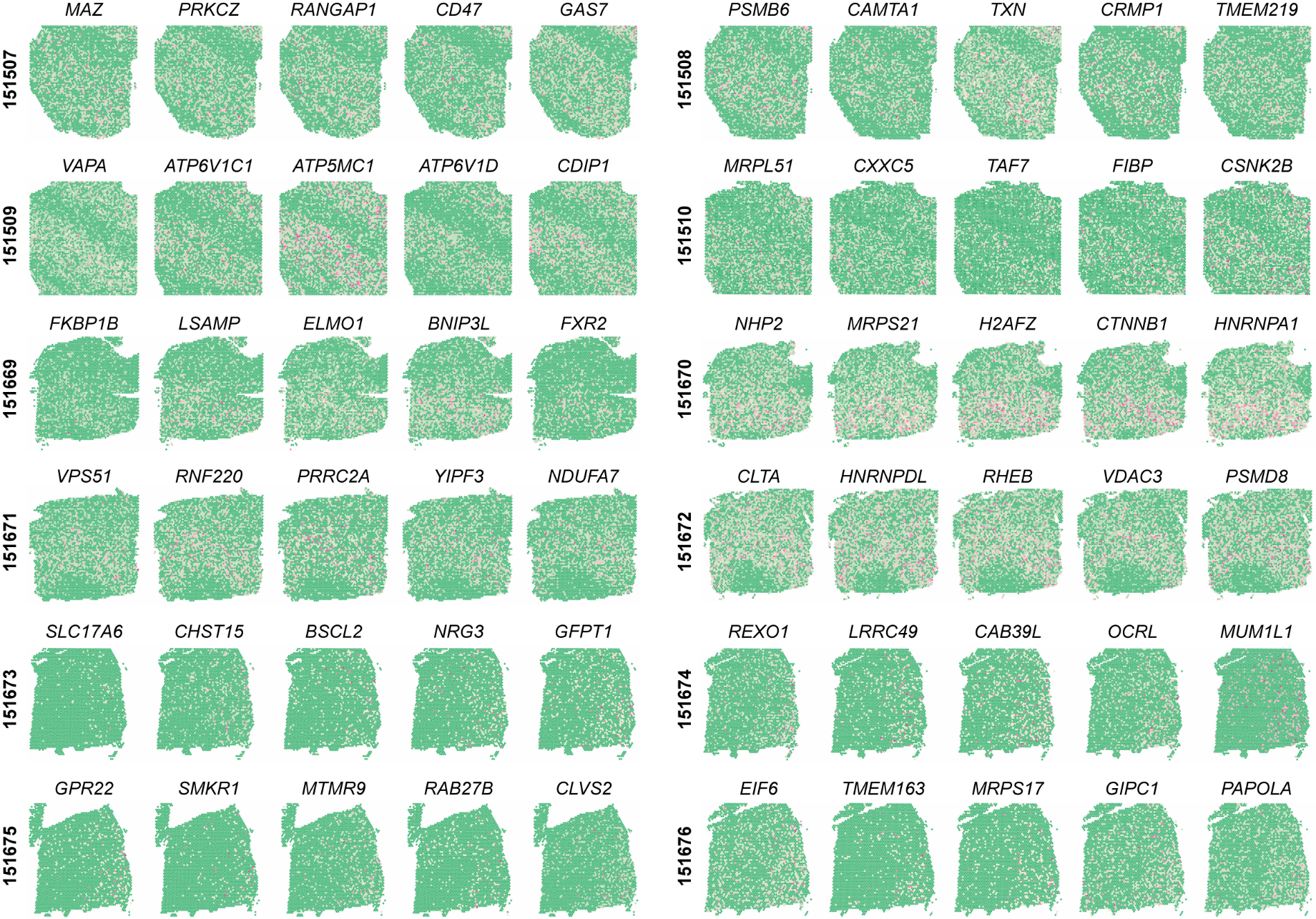
**

**Figure S18. FDPp-Agg enhances the identification of method-specific SVGs.** Visualization of gene expression for SVGs co-identified by FDPp-Agg and individual benchmark methods across twelve DLPFC samples.


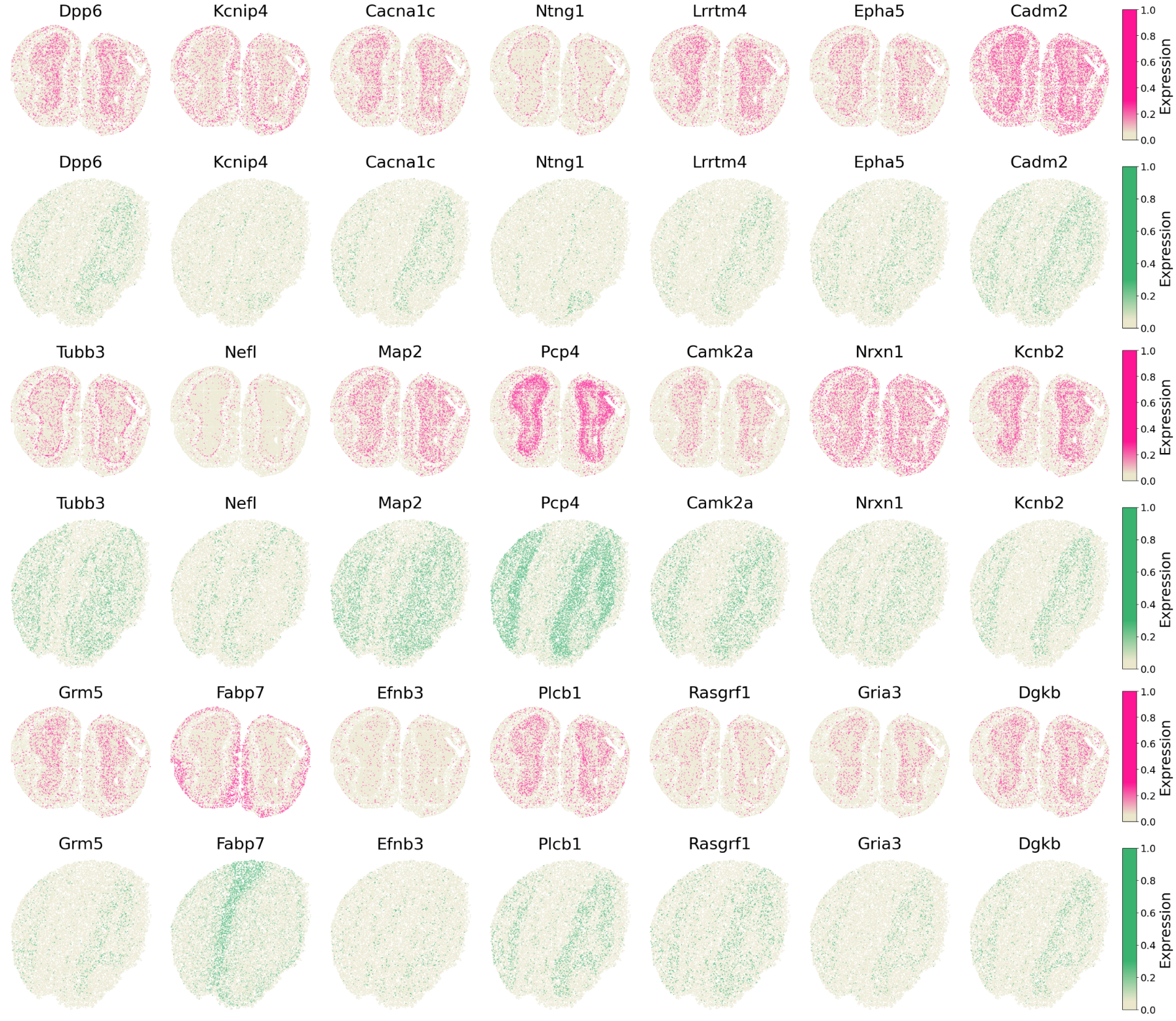


**Figure S19. Castl enables cross-technology consistent identification of laminar SVGs in the mouse olfactory bulb dataset.** Visualization of gene expression for SVGs with significant laminar organization consistently identified by Castl across Stereo-seq (top) and SlideV2-seq (bottom) technologies. Each panel represents a gene.


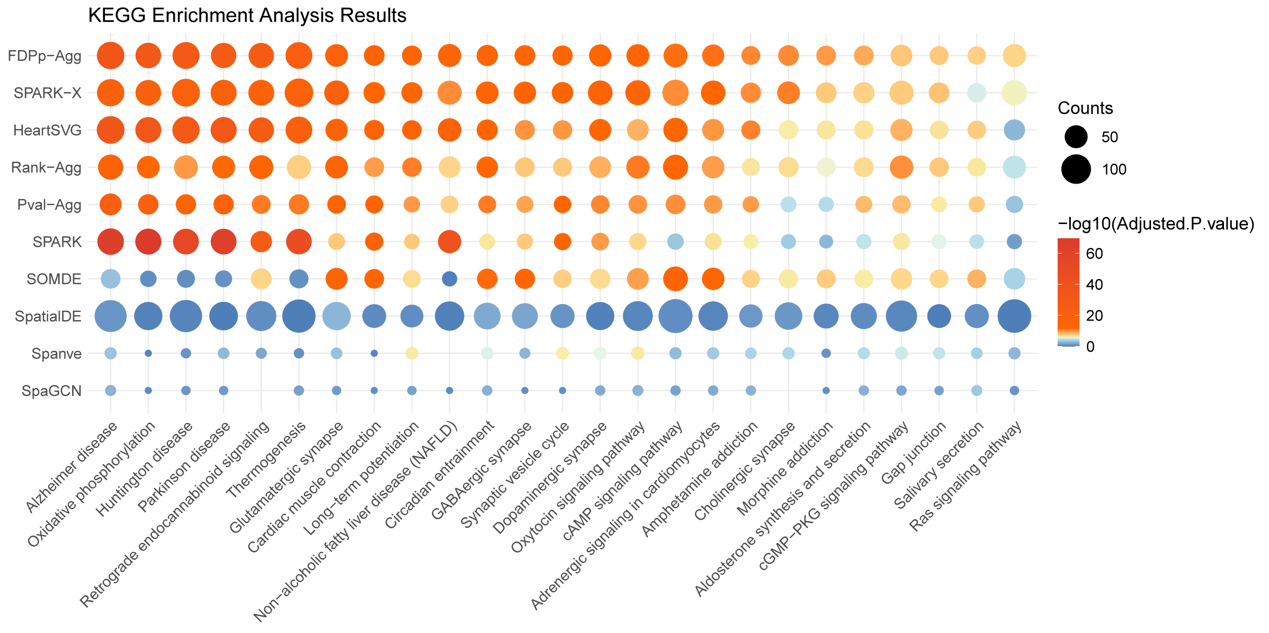


**Figure S20. KEGG enrichment comparison analysis of methods in the mouse olfactory bulb dataset.** The dot plots display the KEGG enrichment of SVGs identified by different detection methods (*y*-axis) in key pathways (*x*-axis) within mouse olfactory bulb datasets generated by SlideV2-seq. The size of each dot represents the intersection magnitude between SVGs identified by the method and enriched genes in the corresponding KEGG pathway, while the color indicates the significance level (-log10(adjusted *p*-value)) of the method in the respective KEGG pathway.


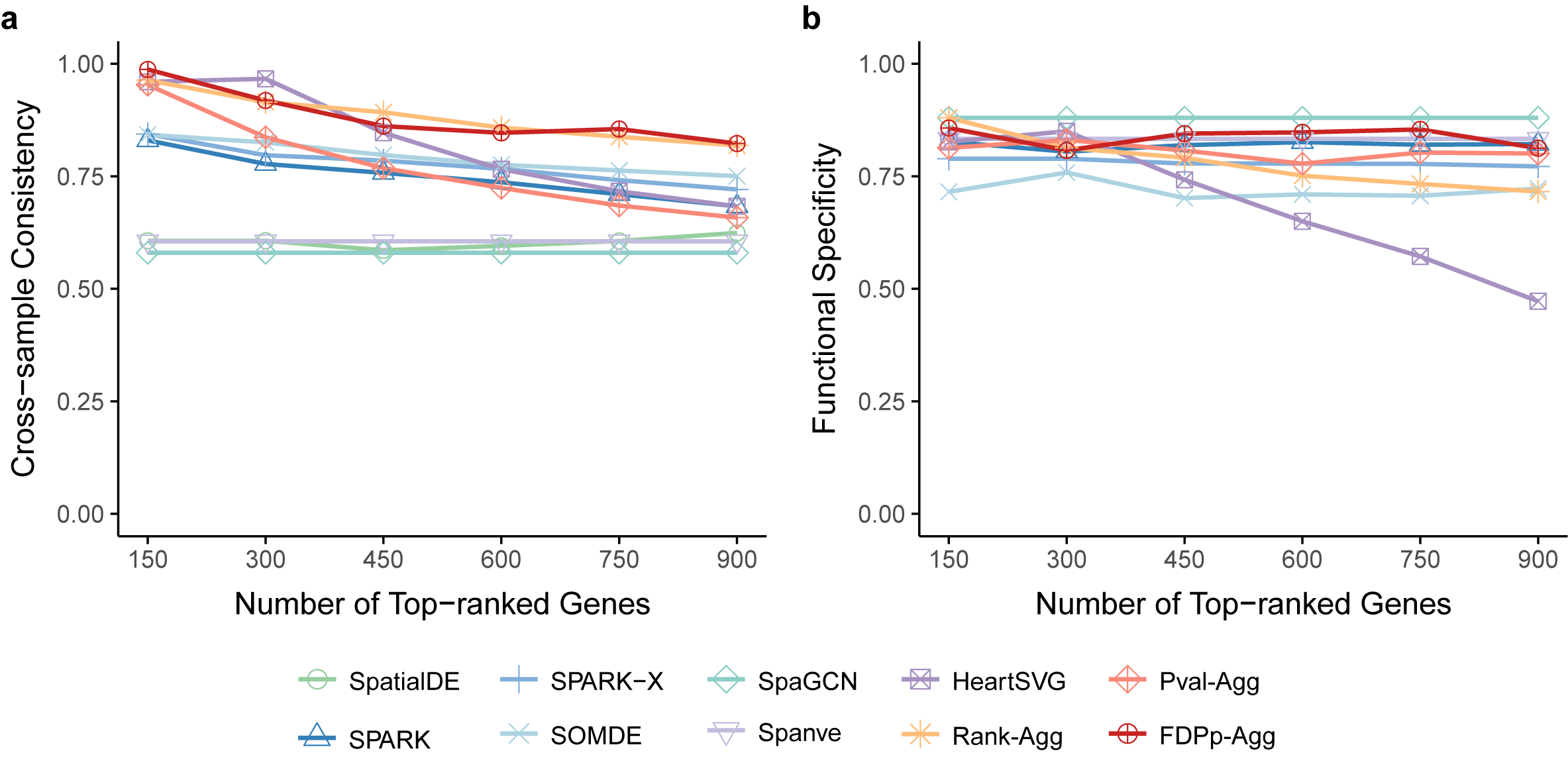


**Figure S21. Detection consistency and functional specificity analysis of methods in the mouse olfactory bulb dataset.** **a** The line plots illustrate the consistency (*y*-axis; see Methods for details) of detection methods across cross-technology datasets for the top 150 to 900 SVGs (*x*-axis), with all identified SVGs used when the number exceeds the method’s detection capacity. **b** The line plots depict the proportion of enriched genes (*y*-axis) among the top 150 to 900 SVGs (*x*-axis) identified by detection methods in cross-technology datasets.


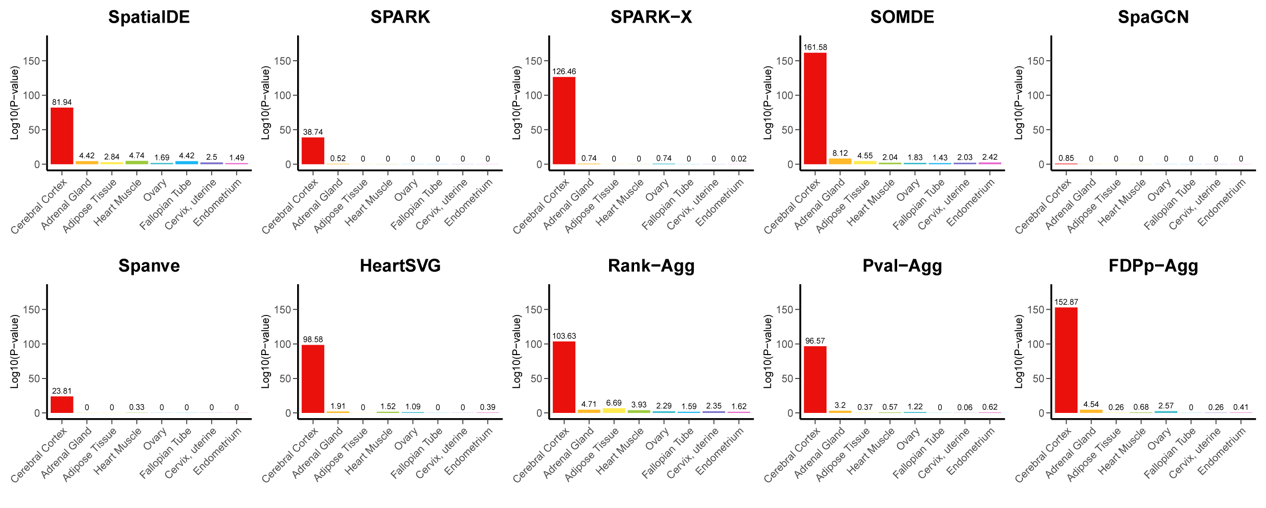


**Figure S22. Tissue-specific enrichment analysis of methods in the mouse olfactory bulb dataset.** The histograms illustrate the enrichment significance (*y*-axis, -log10(*p*-value)) of SVGs identified by different detection methods across various tissues (*x*-axis) in mouse olfactory bulb datasets generated by SlideV2-seq. Each panel represents a distinct detection method.


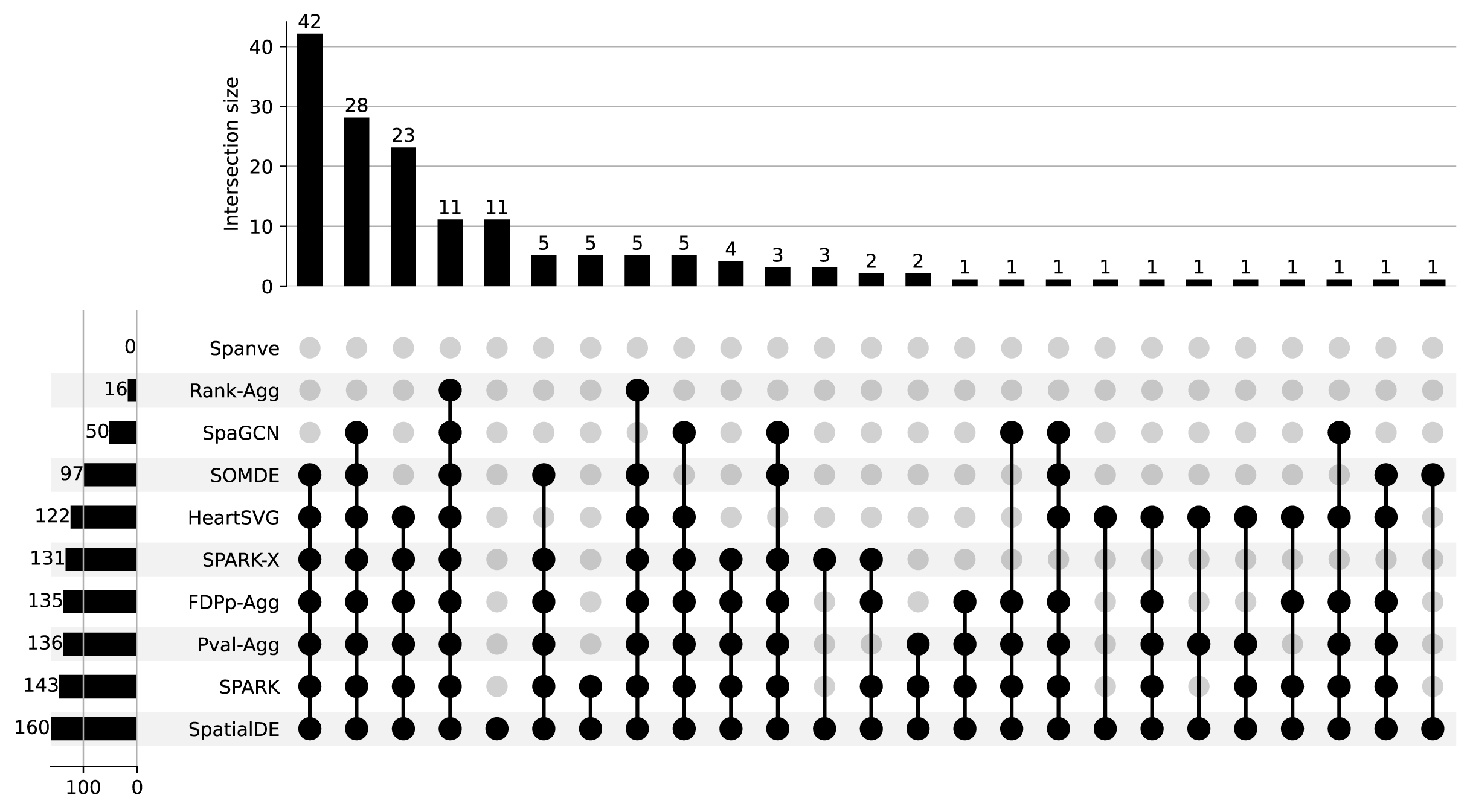


**Figure S23. Upset plots of SVGs identified by different detection methods in the Mouse Hypothalamic Preoptic Region dataset generated by MERFISH.** The upset plot visualizes the intersections of SVGs detected by distinct methods. Rows represent different detection methods, connecting nodes denote specific method combinations, and vertical bar charts correspond to the number of shared SVGs across each combination.


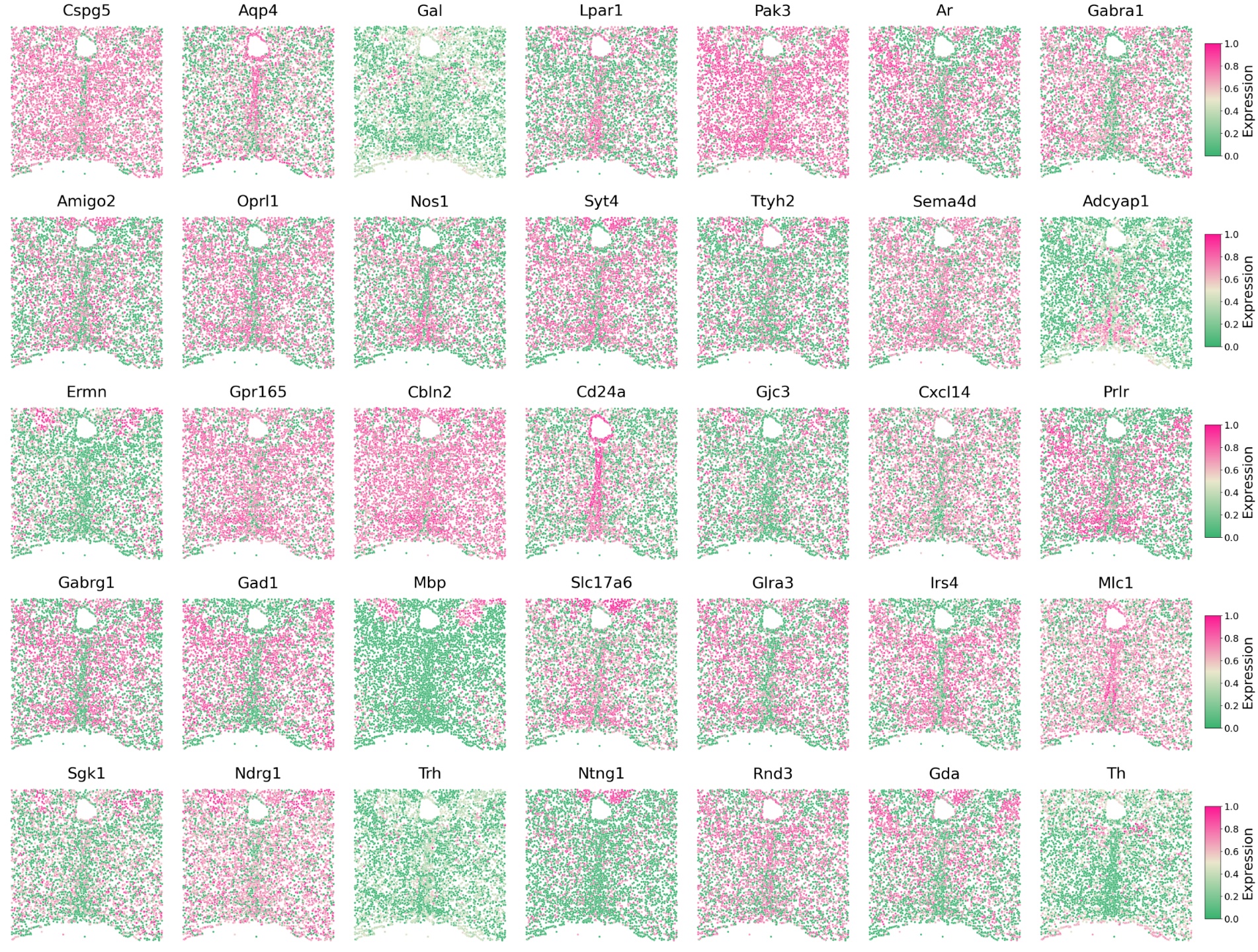


**Figure S24. FDPp-Agg enables cell-type-specific SVGs in the mouse Hypothalamic Preoptic Region dataset.** Gene expression visualization of SVGs identified by FDPp-Agg, which are highly associated with cell types. Each panel represents a single gene.

**References**

1. Svensson V, Teichmann SA, Stegle O. SpatialDE: identification of spatially variable genes. *Nat Methods* 2018;**15**:343-346. https://doi.org/10.1038/nmeth.4636

2. Sun S, Zhu J, Zhou X. Statistical analysis of spatial expression patterns for spatially resolved transcriptomic studies. *Nat Methods* 2020;**17**:193-200. https://doi.org/10.1038/s41592-019-0701-7

3. Zhu J, Sun S, Zhou X. SPARK-X: non-parametric modeling enables scalable and robust detection of spatial expression patterns for large spatial transcriptomic studies. *Genome Biol* 2021;**22**:184. https://doi.org/10.1186/s13059-021-02404-0

4. Hao M, Hua K, Zhang X. SOMDE: a scalable method for identifying spatially variable genes with self-organizing map. *Bioinformatics* 2021;**37**:4392-4398. https://doi.org/10.1093/bioinformatics/btab471

5. Hu J, Li X, Coleman K. *et al*. SpaGCN: Integrating gene expression, spatial location and histology to identify spatial domains and spatially variable genes by graph convolutional network. *Nat Methods* 2021;**18**:1342-1351. https://doi.org/10.1038/s41592-021-01255-8

6. Cai G, Chen Y, Chen S. *et* *al*. Spanve: A Statistical Method for Detecting Downstream-Friendly Spatially Variable Genes in Large-Scale Spatial Transcriptomic Data. *Genom Proteom Bioinf* 2025:qzaf111. https://doi.org/10.1093/gpbjnl/qzaf111

7. Yuan X, Ma Y, Gao R. *et al*. HEARTSVG: a fast and accurate method for identifying spatially variable genes in large-scale spatial transcriptomics. *Nat Commun* 2024;**15**:5700. https://doi.org/10.1038/s41467-024-49846-1

8. Wu Y, Yang S, Ma J. *et al*. Spatiotemporal Immune Landscape of Colorectal Cancer Liver Metastasis at Single-Cell Level. *Cancer Discov* 2022;**12**:134-153. https://doi.org/10.1158/2159-8290.CD-21-0316

9. Maynard KR, Collado-Torres L, Weber LM. *et* *al*. Transcriptome-scale spatial gene expression in the human dorsolateral prefrontal cortex. *Nat Neurosci* 2021;**24**:425-436. https://doi.org/10.1038/s41593-020-00787-0

10. Fan Z, Chen R, Chen X. SpatialDB: a database for spatially resolved transcriptomes. *Nucleic Acids Res* 2020;**48**:D233-D237. https://doi.org/10.1093/nar/gkz934

11. Chen A, Liao S, Cheng M. *et* *al*. Spatiotemporal transcriptomic atlas of mouse organogenesis using DNA nanoball-patterned arrays. *Cell* 2022;**185**:1777-1792.e21. https://doi.org/10.1016/j.cell.2022.04.003

12. Xu H, Fu H, Long Y. *et* *al*. Unsupervised spatially embedded deep representation of spatial transcriptomics. *Genome Med* 2024;**16**:12. https://doi.org/10.1186/s13073-024-01283-x

13. Stickels RR, Murray E, Kumar P. *et* *al*. Highly sensitive spatial transcriptomics at near-cellular resolution with Slide-seqV2. *Nat Biotechnol* 2021;**39**:313-319. https://doi.org/10.1038/s41587-020-0739-1

14. Moffitt JR, Bambah-Mukku D, Eichhorn SW. *et* *al*. Molecular, spatial, and functional single-cell profiling of the hypothalamic preoptic region. *Science* 2018;**362**:eaau5324. https://doi.org/10.1126/science.aau5324

15. Chen KH, Boettiger AN, Moffitt JR. *et al*. RNA imaging. Spatially resolved, highly multiplexed RNA profiling in single cells. *Science* 2015;**348**:aaa6090. https://doi.org/10.1126/science.aaa6090
